# Genome-wide expression profiling and bioinformatics analysis of diurnally regulated genes in the mouse prefrontal cortex

**DOI:** 10.1186/gb-2007-8-11-r247

**Published:** 2007-11-20

**Authors:** Shuzhang Yang, Kai Wang, Otto Valladares, Sridhar Hannenhalli, Maja Bucan

**Affiliations:** 1 Department of Genetics and Penn Center for Bioinformatics, University of Pennsylvania, Philadelphia, PA 19104, USA

## Abstract

**Background:**

The prefrontal cortex is important in regulating sleep and mood. Diurnally regulated genes in the prefrontal cortex may be controlled by the circadian system, by sleep:wake states, or by cellular metabolism or environmental responses. Bioinformatics analysis of these genes will provide insights into a wide-range of pathways that are involved in the pathophysiology of sleep disorders and psychiatric disorders with sleep disturbances.

**Results:**

We examined gene expression in the mouse prefrontal cortex at four time points during a 24 hour (12 hour light:12 hour dark) cycle using microarrays, and identified 3,890 transcripts corresponding to 2,927 genes with diurnally regulated expression patterns. We show that 16% of the genes identified in our study are orthologs of identified clock, clock controlled or sleep/wakefulness induced genes in the mouse liver and suprachiasmatic nucleus, rat cortex and cerebellum, or *Drosophila *head. The diurnal expression patterns were confirmed for 16 out of 18 genes in an independent set of RNA samples. The diurnal genes fall into eight temporal categories with distinct functional attributes, as assessed by Gene Ontology classification and analysis of enriched transcription factor binding sites.

**Conclusion:**

Our analysis demonstrates that approximately 10% of transcripts have diurnally regulated expression patterns in the mouse prefrontal cortex. Functional annotation of these genes will be important for the selection of candidate genes for behavioral mutants in the mouse and for genetic studies of disorders associated with anomalies in the sleep:wake cycle and circadian rhythm.

## Abstract 2

Microarray analysis shows that approximately 10% of transcripts in the mouse prefrontal cortex have diurnally regulated expression patterns.

## Background

The prefrontal cortex is a brain region important for executive functions, including self-observation, planning, prioritizing and decision-making, which are, in turn, based upon more basic cognitive functions, such as attention, working memory, temporal memory and behavioral inhibition [1,2]. The prefrontal cortex is involved in emotional regulation [3] and it also mediates normal sleep physiology, dreaming and sleep-deprivation phenomena. Previous studies show that the prefrontal cortex is particularly sensitive to the negative effects of sleep deprivation, and it benefits the most from sleep [4,5]. In addition, alterations in prefrontal cortex and its connections to other brain regions have been associated with psychiatric disorders (reviewed in [6-8]), including schizophrenia [9], bipolar disorder [10], and attention-deficit/hyperactivity disorder [11].

The pathophysiology of psychiatric and neurodevelopmental disorders, including depression, bipolar disorder, schizophrenia and autism, has been reported to involve disturbances in the sleep:wake cycle and circadian rhythm [12-15]. Both the sleep:wake cycle and circadian rhythms are accompanied by diurnally regulated gene expression - the gene expression levels change daily according to the time of a day. Genome-wide microarray analysis has been used to identify genes with cyclic expression patterns at different circadian time points in the mouse suprachiasmatic nucleus (SCN) and liver using Affymetrix U74A arrays that contain about 10,000 known genes and expressed sequence tags [16] as well as in other mouse tissues, including heart [17] and aorta [18], or in fly heads [19-24]. In addition, sleep/wakefulness regulated genes were studied in the whole cortex, cerebellum, basal forebrain, and hypothalamus in the rat [25,26] and the mouse [27], and in fly heads [24,28,29]. However, these studies assayed only limited numbers of genes, and were focused on either circadian genes (under constant darkness) or tissues other than the prefrontal cortex. Therefore, genome-wide analysis of genes with diurnally regulated expression patterns in the prefrontal cortex will shed light on the function of prefrontal cortex and provide candidate genes for genetic studies of sleep and psychiatric disorders.

In this study, we performed a genome-wide survey of genes with diurnally regulated expression patterns in the mouse prefrontal cortex, a brain region that has not been extensively studied before. In contrast to previous genome-wide studies, which focused on either circadian or homeostatic sleep regulation, our aim was to identify, on a large scale, genes with diurnal rhythms regardless of the controlling mechanisms. We profiled the gene expression levels at four Zeitgeber time (ZT) points during a single day under regular sleep/wakefulness and light:dark cycles, which will capture most diurnally regulated genes that may have different phases. (Thus, in our study, the term 'diurnal' refers to the presence of a day:night cycle rather than being an antonym of 'nocturnal'). We used Affymetrix Mouse430_v2 microarrays, which represent the most extensive mouse gene expression array to date. A total of 2,927 genes were identified as diurnally regulated in the mouse prefrontal cortex, and 2,458 (84%) of them have not been reported before as circadian genes or sleep/wakefulness regulated genes in other tissues and other organisms. Bioinformatics analysis on the diurnal genes revealed eight temporal clusters, each with distinct patterns of expression variation. Each cluster of the genes was associated with specific biological function and was under similar transcriptional regulation.

## Results

### Identification of diurnally regulated genes in the mouse prefrontal cortex

**Figure 1:**
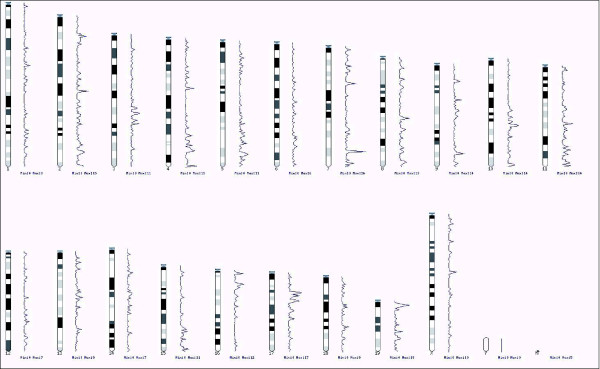
A karyotype map showing the chromosome positions and frequencies of diurnally regulated genes in the mouse genome. Although these genes are scattered around the genome, several regions in chromosomes 7, 17 and 19 show especially high density of diurnally regulated genes.

C57BL/6J mice were entrained to a 12 hour light and 12 hour dark cycle (LD 12:12) for two weeks. We collected tissue samples at four time points, 3 and 9 hours after lights on (ZT3 and ZT9) and 3 and 9 hours after lights off (ZT15 and ZT21), to gain higher resolution temporal patterns of expression and to capture genes whose expression phases would result in similar levels at two time points. To identify genes with diurnally regulated expression levels, RNA samples from the prefrontal cortex of three mice at each ZT point were used for the preparation of cDNA for microarray expression profiling. We expected that examination of gene expression at four time points during the 24 hour light:dark cycle would permit identification of genes regulated by the circadian clock, those controlled by the sleep:wake states, and those induced or suppressed by a wide range of metabolic and environmental conditions. By probing the Affymetrix high-density chip (the Mouse430_v2 array) with approximately 45,000 probe sets, we identified 3,890 probe sets representing 2,927 unique Ensembl genes with diurnally regulated expression levels in the prefrontal cortex at a false discovery rate (FDR) threshold of 20%. We used a relatively liberal FDR threshold because we aimed at identifying a highly comprehensive list of diurnal genes at the cost of decreased specificity. These genes are distributed throughout the mouse genome (Figure 1), and several regions in chromosomes 7, 17 and 19 are especially enriched with diurnally regulated genes.

### Validation of diurnally regulated genes by real-time PCR

**Figure 2:**
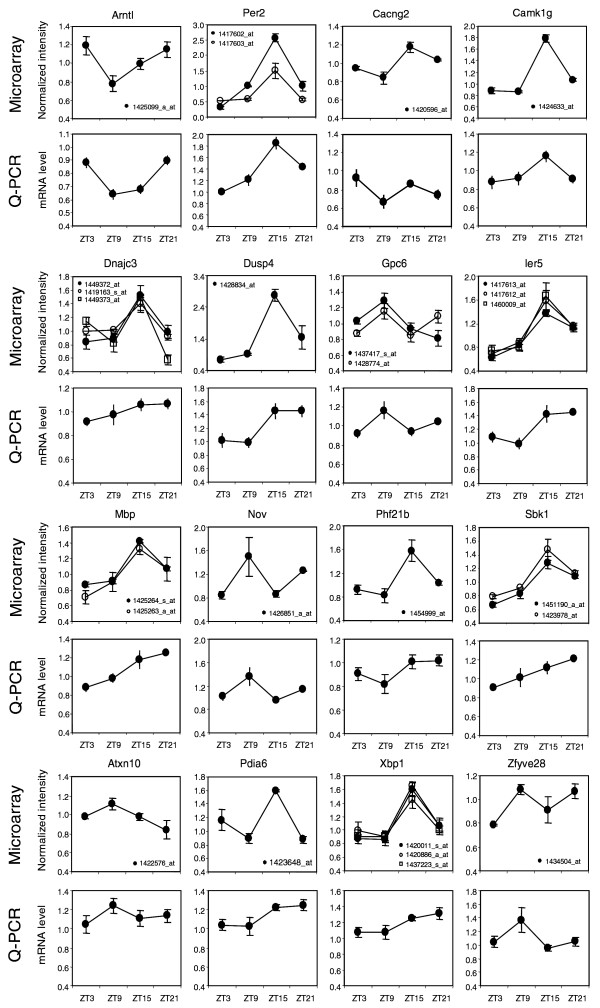
Real-time Q-PCR validation of diurnal genes. For each gene, the expression pattern detected by Q-PCR (lower panel) was compared with that detected by microarray (upper panel). Data shown are mean ± standard error for three biological replicates in the microarrays, and for five biological replicates in Q-PCR experiments. The Q-PCR results gave similar patterns to those detected by the microarray for 16/18 diurnally regulated genes.

To experimentally validate the diurnal expression patterns, we examined the mRNA levels of 18 genes identified in our microarray experiment in independent sets of prefrontal cortex samples (mice) at 4 ZT points by real-time quantitative PCR (Q-PCR). With a motivation to identify candidate genes for neuropsychiatric disorders with sleep anomalies, we selected 12 genes based on the proximity of their human orthologs to previously reported linkage peaks for neuropsychiatric disorders [30,31]. These genes, including *Cacng2*, *Dnajc3*, *Dusp4*, *Gpc6*, *Mbp*, *Nov*, *Phf21b*, *Atxn10*, *Xbp1*, *Zfyve28*, *Rasd2*, and *Sult4a1*, have not been reported to have cycling expression patterns. Several other genes, including *Camk1g*, *Ier5*, *Sbk1*, *Pdia6*, *Bmal1 *(*Arntl*) and *Per2*, were added for additional validation, with *Bmal1 *and *Per2 *serving as positive controls. Of these 18 genes in validation experiments, 16 showed similar or identical patterns to those detected with the microarray experiments (Figure 2), while *Rasd2 *and *Sult4a1 *did not show cycling expression in the Q-PCR experiment (data not shown). Therefore, despite the liberal FDR threshold of 20% in our analysis, we still validated 89% of the diurnal genes that were identified by the microarray experiments. Subtle discrepancies in the expression patterns of several genes between the microarray and Q-PCR results could be due to differences in the oligonucleotide probes on the microarray and the probes used in the Q-PCR experiments.

### Comparison with previously identified cycling genes and sleep/wakefulness related genes

To further validate our data, we compared our list of diurnal genes with a large number of previously described circadian regulated genes and sleep/wakefulness related genes. We queried the Ensembl-Compara database with genes identified in our experiment and genome-wide surveys of cycling genes in the mouse, rat and *Drosophila*. The Ensembl-Compara multi-species database stores the results of genome-wide species comparisons, including ortholog prediction, paralog prediction, whole genome alignments and synteny regions [32]. Although in many cases clear orthologous relationships can not be confidently established, for the 2,927 diurnal genes in the mouse prefrontal cortex, we identified 2,694 human orthologs, 2,810 rat orthologs, and 1,834 *Drosophila *orthologs. Several known core clock genes, such as aryl hydrocarbon receptor nuclear translocator-like (*Arntl *or *Bmal1*), period homolog 1 (*Per1*), period homolog 2 (*Per2*), cryptochrome 1 (*Cry1*), cryptochrome 2 (*Cry2*), basic helix-loop-helix domain containing, class B2 (*Bhlhb2 *or *Dec1*), and genes under circadian control, such as D site albumin promoter binding protein (*Dbp*) and homer homolog 1 (*Homer1*), show diurnal expression in our dataset (Additional data file 1). When we sorted the 2,927 diurnal genes by their FDR q-values (these values represent the significance of expression fluctuation), all of the above genes, except *Arntl *and *Per1*, ranked among the top 522 transcripts, indicating that they encode the most diurnally variable transcripts in the prefrontal cortex. The top 10 genes in this ranking list are heat shock 70 kDa protein 5 (*Hspa5*), myelin basic protein (*Mbp*), calcium/calmodulin-dependent protein kinase IG (*Camk1g*), *Per2*, *Dbp*, splicing factor proline/glutamine rich (*Sfpq*), oxysterol binding protein-like 3 (*Osbpl3*), RanBP-type and C3HC4-type zinc finger containing 1 (*Rbck1*), myeloid/lymphoid or mixed-lineage leukemia 1 (*Mll1*) and Rho family GTPase 2 (*Rnd2*). Among the top ten genes, two (*Per2 *and *Dbp*) are well known circadian genes, four (*Hspa5*, *Rbck1*, *Mll*, *and Rnd2*) have been shown to cycle in mouse SCN and/or other mouse tissues, such as liver, aorta, and kidney [16] (also see their circadian expression patterns in GNF Database of Circadian Gene Expression [33]), *Hspa5 *has been reported as sleep-regulated in rat [25], *Sfpq *has been reported as sleep-regulated in fly [29], and two genes (*Camk1g*, *Mbp*) were validated in our Q-PCR experiments above.

A published survey of 7,000 known genes and 3,000 expressed sequence tags identified approximately 650 cycling transcripts in the mouse liver and SCN [16]. By querying the probe set identifiers against the Ensembl database, we were able to retrieve 759 mouse genes, as well as 608 human, 696 rat and 447 *Drosophila *orthologs, respectively (Table 1). We found 94 common genes in the mouse prefrontal cortex and liver, and 90 common genes in the mouse prefrontal cortex and SCN.

**Table 1:** Orthologous Ensembl genes identified as diurnally regulated in our study or as circadian/sleep:wake controlled in five different studies

	Number of unique genes			
	
Study	Human	Mouse	Rat	*Drosophila*
This study	2,694	**2,927**	2,810	1,834
Panda *et al*. [16]	608	**759**	696	447
Cirelli *et al*. [25]	1,011	1,040	**1,134**	772
Terao *et al*. [26]	105	106	**108**	52
Cirelli *et al*. [24]	228	235	217	**246**
Zimmerman *et al*. [29]	288	318	332	**288**
Total unique genes	4,240	4,628	4,521	2,888

A more detailed list is available in Additional data file 1. For each study, the count of genes in the experimental organism is labeled in bold font.

To examine the representation of sleep- and wakefulness-induced genes among 2,927 diurnal genes in the prefrontal cortex, we integrated previously published data by assigning Ensembl identifiers to genes from these studies. For example, by probing 24,000 rat genes and expressed sequence tags (the rat RGU34A arrays), 752 (4.9%) of the transcripts in the whole cortex and 223 (4.8%) of the transcripts in the cerebellum were identified as regulated by sleep/wakefulness independent of time of day by Cirelli *et al*. [25]. We searched their probe set identifiers against the Ensembl database, and identified 1,053 rat genes as well as 920 human, 962 mouse and 689 *Drosophila *orthologs (Table 1). By comparing our list of mouse diurnal genes with the mouse orthologs of the genes reported by Cirelli *et al*. [25], we found 75 common genes in the sleep-related cortex, 124 in the wakefulness-related cortex, 32 in the sleep-related cerebellum and 67 in the wakefulness-related cerebellum. This significant overlap provides validation for the enrichment of sleep and wakefulness induced genes in the set of diurnal genes over the 24 hour cycle (*P *= 9.2E-49 by one-sided Fisher's exact test). In addition, another similar study examined a small set of 1,200 rat transcripts to identify up- and down-regulated genes in the basal forebrain, cerebral cortex and hypothalamus from rat with sleep deprivation (SD) or recovery sleep (RS) [26]. For this study, we identified 105 human orthologs, 106 mouse orthologs, 108 rat genes and 52 *Drosophila *orthologs from the Ensembl database that are related to sleep/wakefulness (Table 1). We compared our list of diurnal genes in mouse prefrontal cortex with the mouse orthologs of their rat genes, and found 16 (out of 55) common genes that are up-regulated in SD rats, 3 (out of 25) down-regulated in SD rats, 8 (out of 23) up-regulated in RS rats and 5 (out of 26) down-regulated in RS rats. Our list of diurnal genes is enriched for SD and RS related genes (*P *= 0.001 by one-sided Fisher's exact test).

In addition, rest/wakefulness induced genes have also been identified in *Drosophila *[24,29]. From Cirelli *et al*., we retrieved 135 wakefulness related and 14 sleep related *Drosophila *genes with an over 1.5-fold change in expression levels, as well as 136 differentially expressed genes at 4 am, a time when flies are mostly asleep, and 4 pm, a time when flies are mostly awake. We examined mouse orthologs for these genes in our list of diurnal genes in the mouse, and found 19 wakefulness-related genes, 1 sleep-related gene and 16 differentially expressed genes at 4 am and 4 pm in our list. A recent study investigated gene expression changes in the *Drosophila *brain during sleep and during a prolonged period of wakefulness [29]. We retrieved 288 genes from the 252 probe set identifiers in this study that differ in their expression in sleep-deprived *Drosophila *and the control group. We identified 318 mouse orthologs for these genes and found that 63 genes overlap with our diurnal genes list, indicating that our list is enriched for SD related genes (*P *= 6.9e-7 by one-sided Fisher's exact test).

In summary, the above comparative analysis with previous publications revealed 469 diurnal genes that have been reported to be circadian clock related or sleep/wakefulness related. This indicates that a list of 2,458 mouse diurnal genes in our study represent novel findings, mainly due to our unique use of high-density arrays containing approximately 45,000 probe sets and the unique tissue (prefrontal cortex) examined. Despite the liberal FDR threshold used in our study, some of these genes may serve as candidates for studying the role of prefrontal cortex in the regulation of circadian rhythm, diurnal activity and sleep:wake cycles. By assigning Ensembl identifiers for mouse genes with diurnal expression in the prefrontal cortex (this study), mouse genes with cycling expression in the liver and SCN, and four sets of sleep or wakefulness induced genes in the rat and fly, we permit a large-scale comparison of findings performed on different model organisms (Additional data file 1).

### Functional analysis of eight temporal categories of gene expression patterns

**Figure 3:**
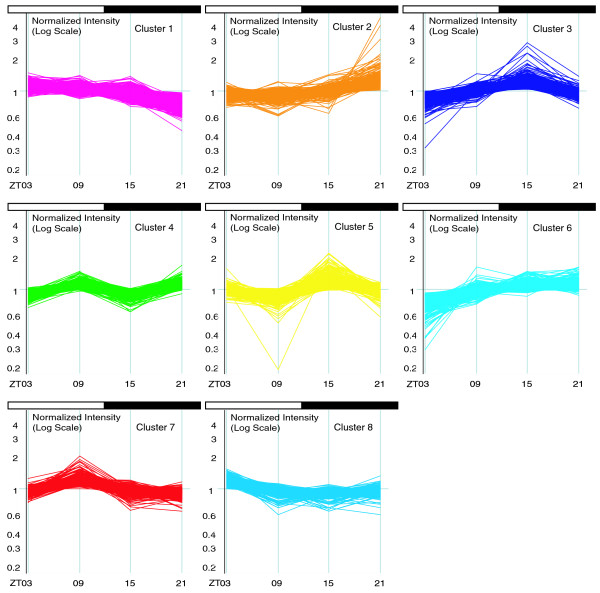
Plots of expression level in log scale versus four time points for the 3,890 diurnally regulated transcripts arranged in eight clusters. These clusters have very distinct temporal patterns of expression variation, suggesting that the clustering procedure is effective in picking out signals specific to each cluster.

The expression levels at four ZT points over a 24 hour cycle allowed us to investigate groups of genes with similar expression patterns, so-called temporal categories. We clustered 3,890 diurnal transcripts in the mouse prefrontal cortex into eight clusters using the K-means clustering algorithm (Figure 3). The clusters each contain from 316 to 698 transcripts, with a distinct pattern of expression and with clearly defined peaks and troughs.

Examination of the eight temporal categories permits several preliminary observations. For example, to identify which clusters are most related to sleep/wakefulness regulation, we examined the overlap of genes in each cluster and the entire set of 1,536 sleep-related genes (combined list of mouse orthologs of genes reported in [24-26,29]) and found that cluster 3 (18.8%) and cluster 5 (21.4%) contain the highest fraction of sleep-related genes.

To investigate whether or not the clustering of diurnal genes correlates with functional groupings, we performed Gene Ontology (GO) functional enrichment analysis on all of the diurnal genes as a whole, and on each cluster of temporally co-expressed genes separately. The GO annotation system uses a controlled and hierarchical vocabulary to assign function to genes or gene products in any organism [34]. Among the three independent GO categories (Biological process (BP), Molecular function (MF) and Cellular component), we focused on the annotation of BP and MF.

Initially, we examined the enrichment of the GO level 3 functional annotations for all of the diurnal genes, using all the genes on the microarray as the background distribution (Table 2). The GO level 3 annotations assign general and broad annotations to genes and gene products, so focusing on this level of annotation reduces multiple testing issues while achieving detailed insights and hints on gene function. Not surprisingly, almost all of the enriched BP categories relate to metabolism, cellular transport/localization and response to stimuli. The enriched MF categories are more heterogeneous, but many of them are related to nucleotide binding, RNA binding or protein binding.

**Table 2:** The most over-represented level 3 GO annotations in the Biological process and Molecular function categories for diurnally regulated genes, using all genes on the Mouse430_2 array as the background distribution

Level 3 GO annotation	Count	*P *value	FDR (%)
**Biological process**			
Macromolecule metabolism	776	1.6e-9	0
Primary metabolism	1,190	4.4e-8	0
Cellular metabolism	1,217	4.6e-6	0
Response to unfolded protein	24	9.4e-5	0.1
Protein localization	155	2.6e-4	0.3
Cell organization and biogenesis	343	5.6e-4	0.7
Cellular localization	140	5.0e-3	6.5
Response to heat	10	1.3e-2	15.8
			
**Molecular function**			
Purine nucleotide binding	343	9.3e-7	0
Transferase activity, transferring phosphorus-containing groups	232	2.9e-6	0
RNA binding	120	8.7e-6	0
Ligase activity, forming carbon-nitrogen bonds	74	4.4e-5	0.1
Unfolded protein binding	39	1.1e-3	1.5
GTPase activator activity	34	1.2e-2	15.2
Protein kinase regulator activity	20	1.4e-2	17.2
Heat shock protein binding	16	1.5e-2	18.1

We next examined enriched GO level 4 annotations for each of the eight clusters of diurnal genes, using all diurnal genes as the background distribution (Table 3). Compared to the analysis using all diurnal genes together, this analysis allowed us to correlate the clusters of temporal categories to more specific functional and biological roles. We found that the eight clusters have a distinct distribution of BP functional categories, suggesting that the clustering results are biologically meaningful. Many of the enriched BP functional categories correspond to specialized aspects of metabolism and cellular responses or the regulation of these processes. For example, genes involved in protein transport and localization are enriched in cluster 1, in which the genes are highly expressed during the rest phase (light phase). Genes responsible for vitamin metabolism are enriched in cluster 6, in which the genes are highly expressed during the active phase (dark phase), when the mice consume most of their food. In cluster 7, where genes have peak expression levels around ZT9 (late in the rest phase), the enriched genes are responsible for the generation of precursor metabolites and energy, which is in preparation for the onset of the active phase. While in cluster 8, genes have higher expression early in the rest phase (ZT3) and are enriched for cellular and macromolecular biosynthesis, consistent with the notion that the sleep phase is important for protein synthesis [25]. Most of the eight clusters do not show clear enrichment of MF functional categories, indicating that each cluster tends to contain genes with different functional roles, but coordinated together in the same biological process. However, it is worth noting that cluster 5, a cluster with higher expression levels early in the active phase (ZT15), is enriched for genes involved in regulating ion channel activity and response to protein stimulus. This may indicate that higher levels of neuronal activities occur during the active phase.

**Table 3:** The most over-represented level 4 GO annotations in the Biological process and Molecular function categories for each of the eight clusters of diurnal genes, using all diurnal genes as background distribution

Cluster	GO level 4 annotation	Count	*P *value	FDR (%)
**Biological process**				
1	Protein transport	49	1.6e-4	0.2
	Establishment of protein localization	49	2.7e-4	0.4
	Intracellular transport	44	1.3e-3	1.8
	Establishment of cellular localization	44	1.8e-3	2.4
	Cellular localization	44	1.8e-3	2.4
	Vesicle-mediated transport	25	1.2e-2	15
2	M phase	12	3.0e-3	4.1
3	Phosphorus metabolism	44	3.4e-4	0.5
	Biopolymer metabolism	97	1.9e-3	2.5
4	None			
5	Response to protein stimulus	8	5.3e-3	7.2
6	Vitamin metabolism	7	1.5e-2	18.6
7	Generation of precursor metabolites and energy	20	1.3e-2	16.6
8	Cellular biosynthesis	25	7.0e-3	9.3
	Antigen processing	4	9.2e-3	12.0
	Macromolecule biosynthesis	17	1.7e-2	21.4
				
**Molecular function**				
1	Guanyl nucleotide binding	22	1.4e-2	17.0
2	None			
3	None			
4	None			
5	Voltage-gated ion channel activity	11	5.7e-4	0.8
	Alkali metal ion binding	8	5.4e-3	6.9
	Calcium ion binding	19	1.0e-2	12.7
	Ion channel activity	12	2.1e-2	24.9
6	None			
7	None			
8	None			

To investigate the associations of diurnally regulated genes with cellular pathways, we queried the KEGG pathway database using the list of all mouse diurnal genes. We found that these genes are significantly enriched in several pathways, including the MAPK signaling pathway (*P *= 8.2e-4, FDR = 0.01), the gap junction (*P *= 1.2e-3, FDR = 0.015) and focal adhesion (*P *= 7.9e-3, FDR = 0.095). Consistent with our results, it has been previously reported that the components of the MAPK pathway tend to have cycling expression levels [35]. Similarly, it has been demonstrated that cell-cell adhesions also play an important role in maintaining and synchronizing circadian rhythms [36].

### Tissue specific expression analysis for diurnally regulated genes

To gain insights into the tissue specificity of expression levels of diurnally regulated genes, we next examined their expression levels in the GNF GeneAtlas dataset, which contains expression patterns for 36,182 GNF probe sets in 61 mouse tissues [37]. Since these mouse tissues are sampled at one time point, we caution that this analysis reflects only a snapshot of the transcriptome for diurnal genes. We plotted the expression levels of diurnal transcripts in 61 tissues as a heat map, and performed a two-way hierarchical clustering for both the genes and the tissues (Additional data file 2). An estimated 25% of the diurnally regulated transcripts are highly expressed in brain-related tissues, such as cerebral cortex, frontal cortex, hippocampus and cerebellum; another estimated 20% are highly expressed in immune-related tissue, such as T cells, B cells and thymus; and the rest of the diurnal genes are highly expressed in various other tissues. Consistent with previous papers on circadian gene expression [16], our results demonstrate that the diurnal gene expression is usually tissue-specific. In addition, we did not observe obvious differences in patterns of tissue-specific expression among genes across the eight temporal categories (data not shown). This indicates that the transcriptional regulatory mechanisms that separated these eight temporal categories are not tissue-specific. It is important, therefore, to examine whether there are specific transcriptional regulatory mechanisms for each temporal category.

### Transcription factor binding site enrichment in the promoters of diurnally regulated genes

It has been shown that the expression of functionally related genes is regulated by groups of transcription factors (TFs), both spatially and temporally [38-40]. Since similar gene expression patterns within a cluster may be attributed to the presence of similar TF binding sites (TFBSs), we next performed TFBS enrichment analysis on the eight temporal categories. The TFBSs were identified using the phylogenetic footprinting approach, which utilizes the known profile or positional weight matrix (PWM) for each TF and the human-mouse evolutionary conservation [41]. For each cluster, we assessed the enrichment statistics of sites identified for each PWM in the 1 kb upstream regions of the genes within the cluster and identified the most enriched TFBSs (Table 4). Because a single TF often has multiple reported PWMs and also different related TFs have similar PWMs, to remove redundancy, we filtered the enriched PWMs that were similar to a more enriched PWM. For instance, if PWM for TF ATF was more enriched than the PWM for CREB, only ATF was retained because the two PWMs are highly similar to each other. Thus, each enriched TF in our analysis should be interpreted as the representative of a family of TFs with similar binding sites. Except for cluster 7, each cluster contains highly enriched TFBSs for several TF families, and these clusters all have a distinct distribution of enriched TFBSs. Altogether, our analysis indicates that transcriptional mechanisms may underlie the different temporal expression patterns for the eight clusters of diurnal genes.

**Table 4:** TFBS enrichment in each of the eight clusters of diurnal genes

Cluster	PWM ID for TFBS	Fold enrichment	*P *value for enrichment	TF family name
1	M00036	1.345	0	v-Jun
	M00248	1.631	0	Oct1
	M00920	1.119	0	E2F
	M00137	1.439	0.001	Oct1
	M00494	1.614	0.003	STAT6
	M01011	1.579	0.003	HNF1
2	M00411	1.383	0	HNF-4alpha1
	M00712	1.457	0.001	Myogenin
	M00331	1.505	0.003	Lentiviral_TATA
	M00261	1.294	0.004	Olf-1
	M00658	1.392	0.005	PU.1
3	M01066	1.577	0	BLIMP1
	M00731	1.915	0.001	Osf2
	M00655	1.3	0.003	PEA3
4	M00116	1.711	0.005	C/EBPalpha
	M00123	1.39	0.005	c-Myc:Max
5	M00641	1.473	0	HSF
	M00736	1.3	0.002	E2F-1:DP-1
6	M00634	1.355	0	GCM
	M00083	1.284	0.002	MZF1
	M01036	1.223	0.004	COUPTF
	M00069	1.265	0.005	YY1
	M00119	1.282	0.005	Max
7	None			
8	M00762	1.446	0.004	PPAR,_HNF-4,_COUP,_RAR

A more detailed list is available in Additional data file 3.

**Figure 4:**
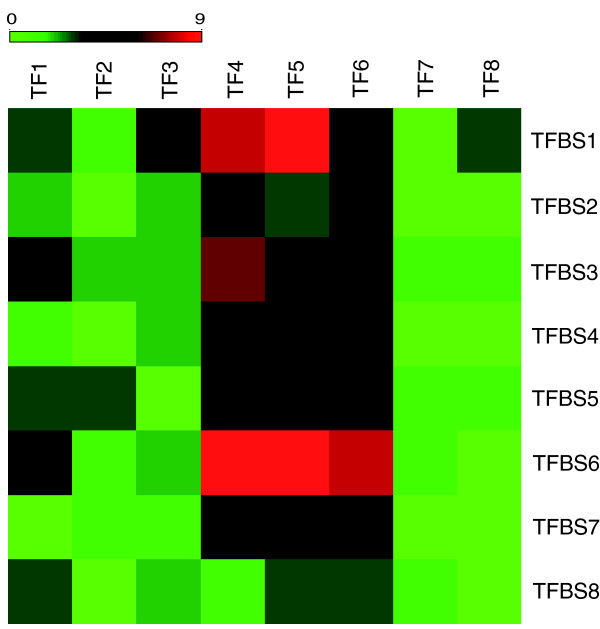
Heat map of enriched TFBSs and their corresponding TFs for each of the eight clusters, when both TFBSs and TFs are present in the diurnal genes. The columns indicate the TFs in each of the eight clusters, where the rows represent the enriched (*P *< 0.05) TFBSs in the 1 kb upstream region of genes in each of the eight clusters. The color of the cell represents the degree of matching: green cells indicate that there is no matching TF and TFBS, while increasing intensity of red colors indicate one or more matches. We found that clusters 2, 7 and 8 contain few TFs that may regulate genes in other clusters, but clusters 4-6 tend to have TFs that may regulate genes in most of the other clusters.

We next examined whether some of the diurnal genes are themselves TFs, and how their corresponding TFBSs are distributed and enriched in the upstream regions of genes in each of the eight clusters. Among the eight clusters, cluster 5 - a cluster enriched with genes involved in response to stimulus - is the most TF-rich cluster, with 19 TFs (with positional weight matrix information in the TRANSFAC database), while cluster 7 - a cluster enriched with metabolism-related genes - is the most TF-poor cluster, with 2 TFs. We generated a heat map to demonstrate the TF-TFBS relationships (Figure 4), and provid detailed statistics on these TFs and TFBSs for each cluster in Additional data file 3. We found that clusters 2, 7 and 8 contain very few TFs whose TFBSs are enriched in other clusters (in Figure 4, cells in the columns for TF1, TF7 and TF8 are mostly green), but cluster 2 and 7 contain many enriched TFBSs that are regulated by TFs in other clusters (in Figure 4, many cells in the rows for TFBS2 and TFBS7 are red). Therefore, the diurnal expression of many genes in these three clusters may be due to the transcriptional control of other clusters. In contrast, clusters 4, 5 and 6 contain TFs whose TFBSs are enriched in many other clusters (in Figure 4, many cells in columns of TF4, TF5 and TF6 are red), indicating that these clusters tend to contain factors that regulate temporal expression of genes in other clusters.

## Discussion

In this study we performed a genome-wide expression profiling analysis on the mouse prefrontal cortex and identified 3,890 transcripts representing 2,927 genes with diurnally regulated expression levels during a 24 hour day:night cycle, among which are 2,458 genes that have not been reported as circadian or sleep:wake related genes in previous studies. Using a clustering analysis, we grouped these diurnal transcripts into categories with similar temporal patterns of expression and showed that these groups differ based on GO functional annotation and distribution of TFBSs in their immediate upstream regions. Annotation of these 2,927 genes will provide a valuable source of candidate genes for behavioral mutations in model organisms such as mouse and for human psychiatric disorders, especially those associated with sleep and circadian disturbances. In addition, annotation of the eight temporal categories can also provide a rich resource for pathway-based functional interpretation of microarray and genome-wide association studies examining cohorts of genes sharing similar functions or co-regulated genes [42].

There are several distinct differences between our study and previous studies on the identification and characterization of oscillating/cycling genes. First, we used the mouse prefrontal cortex as our target for expression profiling, due to its importance in executive functions and in mediating sleep [4,5]. Given the association of psychiatric disorders with malfunctions in prefrontal cortex [9-11], we suggest that diurnal genes in the prefrontal cortex are more likely to be associated with human mental behaviors and psychiatric disorders, particularly those associated with sleep disturbances. As demonstrated in previous experiments, the expression of oscillating genes is highly tissue specific, explaining a small percentage (8.3%) of genes with overlap between SCN and liver in the mouse for circadian genes [16], though the overlap was higher (40-51%) between whole cortex and cerebellum in the rat for wakefulness- and sleep-related genes [25]. It is encouraging that a comparative analysis of our data on mouse prefrontal cortex demonstrated significant (16%) overlap with reports on circadian and sleep/wakefulness related genes. This overlap serves as another means of validation of our findings and further supports previous reports on a subset of genes with cycling expression across tissues.

Second, our goal was to cast a broad net and identify a large number of diurnally regulated genes in a specific tissue, that is, prefrontal cortex. This study does not attempt to distinguish between genes controlled by the circadian system from those regulated by the sleep:wake states. We are aware that a subset of genes identified as diurnally regulated in our study will include genes expressed in response to other external stimuli, including light. This, together with the fact that we used the most extensive arrays and profiled gene expression in a distinct tissue (prefrontal cortex), could explain why most (84%) of the diurnal genes we identified have not been reported in previous circadian and sleep:wake studies in other brain regions from various organisms.

Third, other studies on oscillating gene expression used arrays with relatively few probe sets (less than 10,000 for most publications), but we examined the mouse transcriptome using an array set containing 45,000 probe sets. This large scale analysis enabled us to identify a comprehensive list of genes with diurnal expression levels. Therefore, even though the estimated frequency (approximately 10%) of diurnally regulated genes is similar to previous estimates, the number of genes that we identified is an order of magnitude higher than previous studies. By identifying a large number of diurnally regulated genes in a defined brain region, a clustering analysis resulted in sufficiently large number of genes in each temporal category. There are several main advantages to performing clustering analysis. First, the entire list of diurnal genes may contain genes with many different functions in various cellular pathways. By clustering their patterns of expression variation, we can isolate a specific group of genes with similar expression patterns for more refined functional analysis. For example, analysis of periodically expressed genes in budding yeast showed that genes that encode proteins with a common function often show similar temporal expression patterns, whereas different classes of genes are upregulated at different temporal windows of the respiratory cycles [43]. Second, clustering also allowed us to perform analysis of common sequence motifs and TFBSs on each cluster, which may identify key sequences responsible for common transcriptional regulation. We note that clustering of temporal categories has been performed in several other studies [44,45]. For example, Tavazoie *et al*. [44] used K-means clustering algorithm to cluster 3,000 yeast open reading frames into 30 clusters, based on expression profiles at 15 time points, and subsequently performed functional enrichment analysis and *cis*-regulatory elements analysis. We used the same clustering algorithm to generate eight temporal categories, but used different strategies to analyze the biological meaning of each cluster. We used GO, which is composed of a controlled vocabulary, for the functional enrichment analysis. We also used positional weight matrices from the TRANSFAC database for the TFBS enrichment analysis. Unlike regulatory elements in yeast, the known TFBS profiles in vertebrates are based on experimentally determined binding sites. This, coupled with our use of phylogenetic footprinting to identify putative binding sites, is likely to yield fewer false positives.

We are aware of potential problems and limitations with the current study. We compare gene expression profiles at four time points in a single day rather than sampling tissues over several days. In several similar studies, either a 48 hour period or a 72 hour period was used to study the cycling patterns of expression levels. We acknowledge that sampling more time points over several days would provide more data and higher statistical power for fitting circadian curves; however, our goal in the current study was to identify genes with variable expression levels during the day, rather than genes under circadian control, which requires measurements over a period of several days.

An important aspect of our study is our attempt to establish orthologous relationships between diurnally regulated/cycling genes in different model organisms. This led to the finding that a significant number of brain genes are periodically expressed across species, supporting our prediction that at least a subset of orthologous genes in human will have diurnally regulated expression. We assume that alternations in these genes and even changes in the amplitude of expression due to genetic variation among individuals may contribute to polygenic factors in neurological and psychiatric diseases. Therefore, our study provides a rich source of novel candidate genes and groups of co-regulated genes for human genetic studies. For example, 10 genes among the 16 confirmed genes (*Cacng2*, *Dnajc3*, *Dusp4*, *Gpc6*, *Mbp*, *Nov*, *Phf21b*, *Atxn10*, *Xbp1*, and *Zfyve28*) have their human orthologs located within a 10 Mb region flanking the linkage markers for bipolar disorder [30], and thus merit further study. Prioritization of the list of diurnal genes in mammalian prefrontal cortex by virtue of their chromosomal location in the vicinity of defined susceptibility loci for human neurological and psychiatric disorders and identification of single nucleotide polymorphisms in these genes will represent a first step in this analysis.

## Conclusion

Our analysis demonstrates that about 10% of transcripts have diurnally regulated expression patterns in the mouse prefrontal cortex. These genes can be clustered into eight temporal categories with distinct functional attributes, as assessed by the GO classification and the analysis of enriched TFBSs. Functional annotation of these genes with respect to diurnal expression will be important for the selection of candidate genes for behavioral mutants in model organisms and for human psychiatric disorders, especially those associated with sleep and circadian disturbances.

## Materials and methods

### Animals

All animal experiments were carried out according to the National Institutes of Health guidelines for the use of animals and were approved by the University of Pennsylvania Institutional Animal Care and Use Committee. C57BL/6J mice at ten weeks old were obtained from the Jackson Laboratory (Bar Harbor, ME, USA) and maintained on a LD (12:12) cycle with lights on at 7:00 am. Food and water were available *ad libitum *using standard mouse husbandry procedures. The mice were acclimatized to the lab environment for one week and then entrained for another week under the LD (12:12) cycles with lights on at 7:00 am and 7:00 pm, respectively. We conformed to Zeitgeber time for our experiment, which is used to describe the projected time based on the previous light cycle, with lights on defined as ZT0. At ZT3, ZT9, ZT15, and ZT21, three mice were sacrificed and brains were quickly removed (under red light at ZT15 and ZT21). The prefrontal cortex was defined as described [46] and dissected using the atlas of Franklin and Paxinos as a reference [47]. After removing the olfactory bulb, the most anterior 2 mm cortical area was cut as part of the prefrontal cortex. Then the coronal brain section anterior to the optic chiasm was cut and subcortical structures were removed, which resulted in a tissue about 2 mm ventral from the dorsal surface of the cortex. The prefrontal cortex tissue was put on dry ice immediately after dissection and stored at -80°C until RNA extraction. For validation of the gene expression patterns, we performed the same experiments using another set of mice with five individual animals per ZT.

### Expression profiling experiment

The Affymetrix Mouse430_v2 oligonucleotide microarray (Affymetrix, Santa Clara, CA, USA), which contains 45,037 probe sets, was used for expression profiling experiments. The RNA isolation and the microarray experiment were carried out as described previously [48]. Briefly, total RNA from the mouse prefrontal cortex was isolated using TRIzol reagent (Invitrogen, Carlsbad, CA, USA) followed by cleanup using RNeasy mini kit (Qiagen, Valencia, CA, USA). Total RNA (5 μg) from the prefrontal cortex of each mouse was subjected to cDNA synthesis and each biological replicate was hybridized to one chip, which totals in 12 chips. Microarray data can be accessed through the National Center for Biotechnology Information Gene Expression Omnibus (GEO Series GSE9471).

### Identification and clustering of diurnal genes

Affymetrix Microarray Suite 5.0 was used to quantify expression levels for targeted genes using default parameter values. Probe pairs were scored as positive or negative for detection of the targeted sequence by comparing signals from the perfect match and mismatch probe features. The number of probe pairs meeting the default discrimination threshold (tau = 0.015) was used to assign a call (or flag) of absent, present or marginal for each assayed gene, and a *P *value was calculated to reflect confidence in the detection call. A weighted mean of probe fluorescence (corrected for nonspecific signal by subtracting the mismatch probe value) was calculated using the one-step Tukey's biweight estimate. This signal value, a relative measure of the expression level, was computed for each assayed gene. Global scaling was applied to allow comparison of gene signals across multiple microarrays: after exclusion of the highest and lowest 2%, the average feature signal was calculated and used to determine what scaling factor was required to adjust the chip average to an arbitrary target of 150. All signal values from one microarray were then multiplied by the appropriate scaling factor. The data files were imported to GeneSpring 7 (Silicon Genetics, Redwood City, CA, USA), and to minimize multiple testing problems, the probe list was filtered to include only those that scored as 'present' or 'marginal' in the array software in at least two of the three replicate samples. This resulted in 24,546 probe sets, for which the GCRMA normalized expression values were extracted from the CEL files in GeneSpring 7. The GCRMA normalized data for the 24,546 probe sets were subjected to significance analysis of microarray (SAM) [49] for multiclass analysis of the four ZTs, each with three replicates. Significant genes were selected by adjusting the delta value for a FDR of 20%, and the resulting 3,944 transcripts were further filtered by eliminating genes whose normalized expression levels were lower than 0.9 at all 4 ZTs. The resulting 3,890 probe sets were clustered into 8 groups by their patterns of expression variation, using the K-means unsupervised clustering algorithm implemented in the GeneSpring software. The FDR threshold of 20% is a relatively liberal threshold, because we emphasized the generation of a highly comprehensive gene list over specificity; if the FDR threshold is adjusted to 10%, the number of significant genes drops to 388 and some of the known cycling genes, including *Arntl *and *Per1*, are excluded.

### Validation of diurnally regulated gene expression by real-time Q-PCR

Real-time PCR was carried out on ABI Prism 7900HT sequence detection system (Applied Biosystems, Foster City, CA, USA) by relative quantification (ΔΔCt method) as described previously [48]. Briefly, the total RNA samples isolated from the prefrontal cortex were reverse-transcribed into cDNA using a High Capacity cDNA Archive Kit (Applied Biosystems). The cDNA were then subjected to real-time PCR for 18 target genes (*Arntl*, *Per2*, *Cacng2*, *Camk1g*, *Dnajc3*, *Dusp4*, *Gpc6*, *Ier5*, *Mbp*, *Nov*, *Phf21b*, *Rasd2*, *Sbk1*, *Atxn10*, *Sult4a1*, *Pdia6*, *Xbp1*, and *Zfyve28*) using rodent GAPDH as endogenous control. All the TaqMan assays and reagents were from Applied Biosystems. Three replicates were performed for each of the five mice at ZT3, ZT9, ZT15, and ZT21. Statistical analysis was performed using one-way ANOVA and *t*-test to evaluate expression fluctuations across the four ZTs.

### Comparison of our diurnal genes to genes previously reported

Several other publications reported genes in rats or mice under different environments, such as under regulation of the circadian system or under sleep/wakefulness control. For the previously published experiments, the probe set identifiers were retrieved from the supplementary materials of the publications and translated to Ensembl gene identifiers by querying the Ensembl database (version 42, December 2006). Several previously published data sets were collected on rats or flies, so we queried our mouse diurnal genes against the Ensembl-Compara database [32], and collected the corresponding orthologous genes for comparative analysis. This procedure ensures the most comprehensive and up-to-date translations between the probe set identifiers and gene identifiers.

### Functional analysis of genes with diurnally regulated expression

The DAVID 2007 web server [50] was used for functional analysis of the diurnally regulated genes. When analyzing the common enriched functional categories among the diurnal genes, all genes in the genome were used as the 'background population'; when analyzing each of the eight clusters of diurnal genes, all the diurnal genes were used as the 'background population'. The GO scheme was adopted for functional annotation of diurnal genes, and GO levels of 3 for broader annotations and 4 for specific annotations were used. The *P *values are calculated from one-sided Fisher's exact test. Due to the lack of independence between genes and between GO categories, there has not been a golden-standard way to perform *P *value adjustment for gene enrichment analysis. Therefore, we also provide the FDR measure and caution that the table could contain some false positive GO categories.

### Tissue-specific expression analysis of diurnal genes

We collected the GNF GeneAtlas mouse expression data sets [37] from GNF Genome Informatics Applications and Datasets [51]. This data set contains expression measures for 36,182 GNF probe sets in 61 mouse tissues, and the raw data were processed by the GC-RMA normalization procedure. We used GNF's annotation file to translate these probe set identifiers to Ensembl transcript identifiers, to establish the correspondence with our diurnal transcripts. We were able to retrieve expression measures for 2,097 diurnal transcripts in the GNF data set. We then used the two-way hierarchical clustering algorithm implemented in the Hierarchical Clustering Explorer software [52] to cluster both the genes and the tissues.

### Transcription factor binding site analysis

A phylogenetic-footprinting approach to predict TFBSs in human and mouse was previously reported [41]. Using this approach, a comprehensive mouse TFBS database was built. Briefly, for each gene in the mouse genome, the 1 kb genomic sequence immediately upstream of the transcription start site was searched using the 546 vertebrate PWMs obtained from the TRANFAC database v8.4 [53]. A PWM is a 4 × *k *matrix for a *k *bases long binding site and provides, for each of the *k *positions, the preferences for the four nucleotide bases at that position. Matches between TRANFAC PWMs and promoter regions of the mouse genes were selected using the tool PWMSCAN [41]. The criterion for a match was a *P *value cutoff of 2 × 10^-4^, corresponding to a chance occurrence of one match per 5 kb on average. These matches were filtered further using human-mouse genome sequence alignments to focus our analyses on promoter regions that showed evolutionary conservation. For each TRANSFAC match the fraction *c *of binding site bases that were identical between human and mouse was computed, and the matches for which either *P *value = 0.00002 (expected frequency of 1 in 50 kb) or *c *= 0.8 were retained.

The over-representation of TFBSs in each gene cluster was calculated by dividing the frequency with which a given TFBS was present in promoters of genes in the cluster by its frequency in the promoters of all diurnal genes. Statistical significance was then assessed by permutation tests. More specifically, let *P *denote the set of 1 kb promoter sequences of genes in a given cluster, and let *C *represent the promoter sequences for the entire set of diurnal genes. For each of the 546 transcription factor PWMs, define over-representation of the PWM *x*_*i *_as:

$$s_{i}=\frac{{\text{number of x}}_{\text{i}}\text{ in P}}{{\text{number of x}}_{\text{i}}\text{ in C}}*\frac{\left|C\right|}{\left|P\right|},$$

where |P| and |C| are the number of sequences in P and C, respectively. Let P' be a set of |P| sequences, randomly selected from *C*. Analogous to *s*_*i*_, we calculate over-representation *s*_*i*_' in P' relative to C. Assume that *s*_*i *_= 1. In 1,000 such random samplings, the fraction of times in which the over-representation *s*_*i*_' = *s*_*i *_estimates the significance of *s*_*i*_.

## Abbreviations

BP, Biological process; FDR, false discovery rate; GO, Gene Ontology; MF, Molecular function; PWM, positional weight matrix; Q-PCR, quantitative PCR; RS, recovery sleep; SD, sleep deprivation; TF, transcription factor; TFBS, transcription factor binding site; ZT, Zeitgeber time.

## Authors' contributions

SY performed microarray experiments, real-time PCR validation, raw data processing and clustering analysis. KW and OV conducted bioinformatics analysis on the diurnal genes and comparative analysis with other publications. SH performed transcription factor binding site enrichment analysis. MB conceived the study, guided the interpretation of data and provided intellectual mentorship and guidance. All authors contributed to the writing and approved the final version of the manuscript.

## Additional data files

The following additional data are available with the online version of this paper. Additional data file 1 provides 62 additional tables listing expression values at four time points for diurnally regulated genes, the clustering results from the GeneSpring software, and the gene identifiers used in our comparative analysis with five other publications. Additional data file 2 is a figure illustrating the heat map of expression levels for diurnal genes in 61 mouse tissues, with two-way hierarchical clustering for both the tissues and the genes. Additional data file provides three additional tables listing detailed statistics values as well as TFBS names and identifiers for TFBS enrichment analysis in the eight clusters of genes.

## Supplementary Material

Additional data file 1 Expression values at four time points for diurnally regulated genes, the clustering results from the GeneSpring software, and the gene identifiers used in our comparative analysis with five other publications. Click here for file

Additional data file 2 Heat map of expression levels for diurnal genes in 61 mouse tissues, with two-way hierarchical clustering for both the tissues and the genes. The red rectangular box around tissue names indicates brain-related tissues. Click here for file

Additional data file 3 Detailed statistics values as well as TFBS names and identifiers for TFBS enrichment analysis in the eight clusters of genes. Click here for file
