# Supplementary figures and images for: Genome-wide expression profiling and bioinformatics analysis of diurnally regulated genes in the mouse prefrontal cortex
Source: Genome Biol. 2007 Nov 20;8(11):R247. doi: 10.1186/gb-2007-8-11-r247 (PMC2258187; doi:10.1186/gb-2007-8-11-r247)

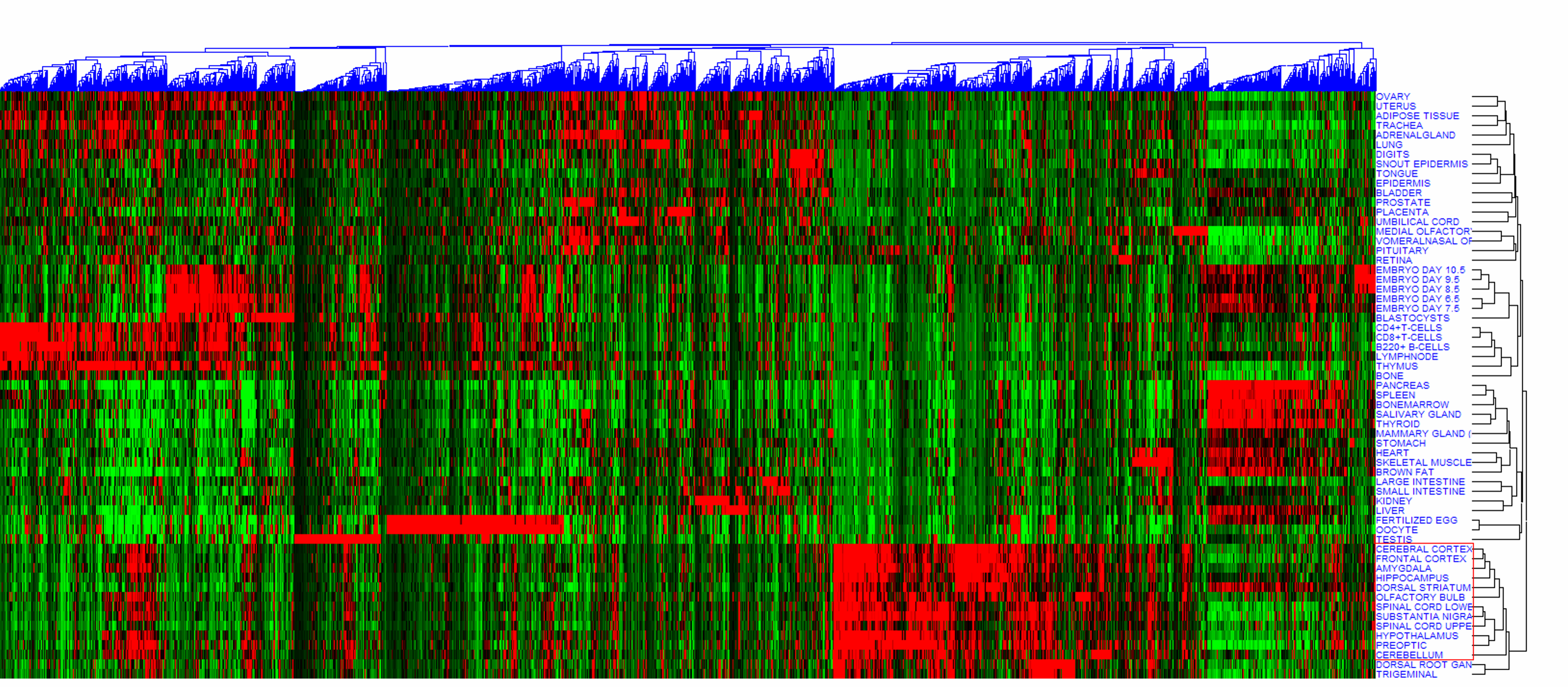

Supplement: Additional data file 2 — Heat map of expression levels for diurnal genes in 61 mouse tissues, with two-way hierarchical clustering for both the tissues and the genes. The red rectangular box around tissue names indicates brain-related tissues. [file gb-2007-8-11-r247-S2.tiff]
